# Gut Microbiome and Serum Metabolome Profiles of Capsaicin with Cognitive Benefits in APP/PS1 Mice

**DOI:** 10.3390/nu15010118

**Published:** 2022-12-27

**Authors:** Jun Li, Xiaojun Liao, Xuedong Yin, Zimeng Deng, Guangfen Hu, Weiwei Zhang, Feng Jiang, Liang Zhao

**Affiliations:** 1 National Engineering Research Center for Fruit & Vegetable Processing, Key Laboratory of Fruit & Vegetable Processing, Ministry of Agriculture and Rural Affairs, Engineering Research Centre for Fruits and Vegetables Processing, Ministry of Education, Beijing Key Laboratory for Food Non-Thermal Processing, College of Food Science and Nutritional Engineering, China Agricultural University, Beijing 100083, China; 2 Guizhou Guisanhong Food Company Limited, Zunyi 563000, China

**Keywords:** capsaicin, Alzheimer’s disease, gut microbiota, *Akkermansia muciniphila*, serum metabolomics, tryptophan metabolism

## Abstract

Capsaicin, a natural bioactive component, has been reported to improve cognition and ameliorate the pathology of Alzheimer’s disease (AD). Studies have linked AD to alterations in gut microbiota composition and serum metabolites. In the present study, we examined the alterations in serum metabolome and gut microbiome in APPswe/PS1dE9 (APP/PS1) mice treated with capsaicin. Capsaicin treatments resulted in a significant increase in the abundance of *Akkermansia*, *Faecalibaculum*, *Unclassified_f_Atopobiaceae*, and *Gordonibacter* and a significant decrease in the abundance of *Adlercreutzia*, *Peptococcaceae*, *Alistipes*, *Oscillibacter* and *Erysipelatoclostridium*. Furthermore, the species *Akkermansia muciniphila* (*A. muciniphila*) was significantly enriched in capsaicin-treated APP/PS1 mice (*p* = 0.0002). Serum metabolomic analysis showed that capsaicin-treated APP/PS1 mice had a significant higher level of tryptophan (Trp) metabolism and a significantly lower level of lipid metabolism compared with vehicle-treated mice. Capsaicin altered serum metabolites, including Kynurenine (Kyn), 5-Hydroxy-L-tryptophan (5-HIT), 5-Hydroxyindoleacetic acid (5-HIAA), indoxylsulfuric acid, lysophosphatidyl cholines (LysoPCs), and lysophosphatidyl ethanolamine (LysoPE). Significant correlations were observed between the gut bacteria and serum metabolite. With regard to the increased abundance of *A. muciniphila* and the ensuing rise in tryptophan metabolites, our data show that capsaicin alters both the gut microbiota and blood metabolites. By altering the gut microbiome and serum metabolome, a diet high in capsaicin may reduce the incidence and development of AD.

## 1. Introduction

Alzheimer’s disease (AD), known as a progressive age-related neurodegenerative disorder, affects over 50 million people worldwide. The number of patients with AD is predicted to increase to 152 million in 2050 [1]. The aggregations of extracellularly β-amyloid (Aβ) plaques and hyperphosphorylated tau proteins as intracellularly neurofibrillary tangles are the featured neuropathological hallmarks [2]. AD patients suffer a decline in memory, cognitive abilities, and behavior, often for many years, which seriously affects patients, families, and the public health system [3]. Although drugs interventions alleviate or reverse the elusively aforementioned AD symptoms, there is no curative or disease-modifying treatment for AD. Prevalent methods for reducing the risk of AD include enhancing physical activity, reducing obesity, and choosing balanced diets.

Numerous studies have proven the relationship between the gut microbiota and the occurrence and development of AD [4]. Qian et al. reported the role of gut microbiota in AD via inflammatory pathways, because there existed a decreasing diversity of gut microbiota in AD patients. Specially, Firmicutes and Actinobacteria decreased, while Bacteroidetes increased at the phylum level in AD [5]. Germ-free (GF) mice have not been naturally colonized by microorganisms and represent a model system for studying the effect of gut microbiota on host physiology. Studies have shown that gut microbiota in GF mice can affect behavior, based on changes brain physiology and neurobiochemistry [6]. GF mice significantly absence of amyloid plaque build-up in a mouse model for Alzheimer’s disease [7]. Transgenic mice treated with an antibiotic cocktail (ABX) reduced amyloid plaques in the hippocampus, because of the significant impact of ABX-mediated alterations in the microbiome [8]. Remodeling the gut microbiota via sodium oligomannate (GV-971) led to the peripheral accumulation of phenylalanine and isoleucine and inhibited AD progression [9].

Diet management has been linked to a reduced risk of several chronic diseases, which mediates gut microbiota structure and functions. Modified Mediterranean-ketogenic diet modulates gut microbiome and metabolites in association with cerebrospinal fluid (CSF) AD biomarkers [10]. Fermentation of dietary fiber is one of the dominant functions of the caecal and colonic microbiota and a major source for SCFAs, which have an important role in maintaining intestinal immune homeostasis and protecting against inflammation [11]. Capsaicin is a natural product isolated from chili pepper, and it is consumed as a vegetable and spice. There is much epidemiological and research evidence linking capsaicin-rich diets with AD incidence. A higher consumption of chili pepper causes mild differences in incidence rates for AD between eastern and western regions [12]. Liu et al. observed that spicy diets improved the cognitive deficits of people that were over 40 years old [13]. Wang et al. found that capsaicin reduced Aβ depositions by boosting the maturation of ADAM10 and shifting the APP processing pathway [14]. Capsaicin alleviates cognitive impairments of the APP23/PS45 mouse model of AD [15]. Dietary capsaicin beneficially alters the composition of the gut microbiota and short-chain fatty acid metabolism against obesity [16]. Capsaicin inhibits the increase in abundance of the genus Lactobacillus and its bile acid metabolism Type 2 diabetic db/db mice [17]. However, few researchers have evaluated the changes in the gut microbiome and serum metabolism by capsaicin administration and their correlation in APP/PS1 mice. Here, we studied the effects of capsaicin on cognition, Aβ plaques, gut microbiota, and serum profile in APP/PS1 mice.

In this research study, we selected the APP/PS1 mouse as a model for AD; in this model, Aβ plaques are observed at the age of 4 months, with visible cognitive deficits with respect to spatial learning at 8 months [18,19]. The APP/PS1 mice were used to evaluate the potential effects of capsaicin treatments on behavioral disorders. Additionally, the effect of capsaicin on the aggregation of Aβ plaques was also determined. Furthermore, 16S rRNA-sequencing and untargeted metabolism analyses were performed to investigate the effect of capsaicin on the gut microbiota and metabolome profiling. Our study demonstrates that capsaicin-rich diets could cause a decline in cognitive disorder, reduce the aggregation of Aβ plaques, alter the structure and composition of gut microbiota, and change the profile of serum metabolomics, thus highlighting the observation that dietary capsaicin is easily implementable for attenuating AD.

## 2. Materials and Methods

### 2.1. Mice

C57BL/6J wild-type (WT) and APPswe/PSEN1dE9 (APP/PS1) male transgenic mice aged 4.5 months old were selected for our study. All animals (SPF Biotechnology Co., Ltd., Beijing, China) were housed in a standard SPF facility of Peking University Health Science Center Department of Laboratory Animal Science. All the mice were maintained in a room at 23 °C under a 12 h (h) light/dark cycle. All mice were fed a standard chow diet and isocaloric diet. APP/PS1 mice were randomly assigned into two groups (*n* = 7 per group), and the WT group included 9 mice. All experimental procedures were approved by the respective Institutional Animal Care and Use Committees of China Agricultural University (Issue number: Aw20902202-4-1). At the end, feces were collected and stored at −80 °C. The cognitive function was assessed. Mice were then sacrificed, and their brain tissues and blood samples were collected.

### 2.2. Gavage Protocol

Mice were gavaged daily for 4 months with capsaicin (Tautochem, Shanghai, China) or vehicle. The characterization of treatments was conducted as follows: WT mice gavaged with vehicles (referred to as WT_CTRL), APP/PS1 gavaged with vehicle (referred to as APP/PS1_CTRL), and APP/PS1 gavaged with 40 mg/kg·day capsaicin (referred to as APP/PS1_CP).

### 2.3. Histology

Brain tissue sections were stained with Congo red to assess Aβ aggregations in the hippocampus and the cortex from mice [20]. In brief, the tissue sections were mounted on slides. The slides were soaked in 4% paraformaldehyde for 12 h and flushed with ddH2O for 2 min. The slides were placed in NaCl-hydroxide-ethanol solution for 20 min and directly transferred in Congo red solution for 20–30 min; then, they were rinsed with ddH2O. After dehydration, the slides were observed with microscope inspections (3DHISTECH, Budapest, Hungary). 

### 2.4. Behavioral Tests

The Morris water maze test was selected to evaluate spatial memory formation and retention [21,22,23]. In brief, a platform was submerged 1 cm below the latex white water surface into a round tank (120 cm in diameter and 50 cm in depth). Four different visual cues were placed on the wall around the pool. The water temperature was kept at 20 ± 2 °C. The procedure included six training days and one probe trial test day. During the training sessions, all mice could swim freely lasting for 60 s or get onto the platform. Otherwise, the researchers placed them on platform and kept them on it for 10 s. During test day, the platform was removed, and the animals were permitted to swim freely for 60 s. All data were acquired and analyzed using the ANY-maze (Stoelting, Chicago, IL, USA) video tracking system. 

The Y maze test assessed spatial working and reference memory in mice. The Y-shaped maze had three light-colored arms (start arm, other arm and novel arm) orientated at 120° angles from each other. During training trials, the researchers placed mice into the start arm and allowed them to wander within two arms for 10 min without the novel arm. After a 1 h interval, the novel arm opened, and the same test mouse was allowed to explore all arms for 5 min. All data were acquired and analyzed using the SuperMaze video tracking system. The number of times the mouse entered to the novel arm and other two arms was recorded.

### 2.5. Fecal Microbiome Preprocessing and Data Analysis

Sample collection, DNA extraction, and 16S ribosomal ribonucleic acid (rRNA) amplicon-based sequencing were included. Briefly, at the end of the experiment, pellet samples were collected. Bacterial genomic DNA was extracted from each fecal sample using the E.Z.N.A.^®^ soil DNA Kit (Omega Bio-tek, Norcross, GA, USA). The amplification of 16S rRNA used the primers that were flanking all V regions ([forward overhang] + 27F: [5′-AGAGTTTGATCCTGGCTCAG-3′]; and [reverse overhang] +1429R: [5′-TACGACTTAACCCCAATCGC-3′]). All samples were sequenced together on a PACBIO_SMRT platform by Majorbio Bio-Pharm Technology Co. Ltd. (Shanghai, China).

Microbiome diversity analyses were performed using QIIME 2 (version 2020.2). The operational taxonomic unit (OTU) table for all samples generated in QIIME 2 was used to assess differences in the relative abundance of microbial genera. Alpha diversity and beta diversity were separately evaluated by the Mothur metric and the Bray–Curtis metric. Moreover, statistical significance was assessed using the Adonis function.

### 2.6. Serum Metabolomics Analyses

Serum samples were cleaned up with methanol protein precipitation and characterized with ultra-high pressure liquid chromatography coupled with high resolution mass spectrometry (UHPLC-MS/MS). Samples measuring 100 μL were mixed with 400 μL methanol–water (4:1, *v/v*) and homogenized for 30 s. The solution was ultrasonically extracted on ice for 30 min (5 °C, 40 KHz) and stored at −20 °C for 30 min; then, the solution was centrifuged at 13,000 rpm for 10 min at 4 °C.

LC-MS/MS analyses were selected using UHPLC System (Agilent Technologies, California, CA, USA), equipped with a ACQUITY UPLC HSS T3 column (100 mm × 2.1 mm i.d., 1.8 μm; Waters, Milford, MA, USA). The volume of one sample was 10 μL and the mobile phases consisted of 5% acetonitrile in water (A) and acetonitrile: isopropanol 1:1 (B), flowing at a rate of 0.4 mL/min. The analysis was carried out with a 16 min gradient: 0–0.5 min, 5–20% B; 0.5–2.5 min, 20–25% B; 2.5–9 min, 25–95% B; 9–13 min, 95% B; 13–13.1 min, 95–5% B; 13.1–16 min, 5% B. The mass spectrometric system was equipped with an electrospray ionization (ESI) source. ESI source conditions were set as follows: source temperature as 550 °C; gas 1 as 50 psi, gas 2 as 50 psi, curtain gas as 35 psi, ion spray voltage floating (ISVF) as 5000–4000 V, declustering potential as 60 V, MS/MS at collision energy (CE) as 30 eV, and cycle time was 0.51 s.

### 2.7. Statistics

All data are expressed as the mean ± standard error of the mean (SEM) and were analyzed using GraphPad Prism Software for Windows. The unpaired two-tailed Student *t*-test and one-way ANOVA followed by Tukey’s multiple comparison test were used for differences between two groups and among more than two groups, respectively. Correlations were analyzed using Spearman’s correlation. The minimum significance value was considered as *p* < 0.05.

## 3. Results

### 3.1. Capsaicin Improved Cognitive Deficts in APP/PS1 Mice

**Figure 1:**
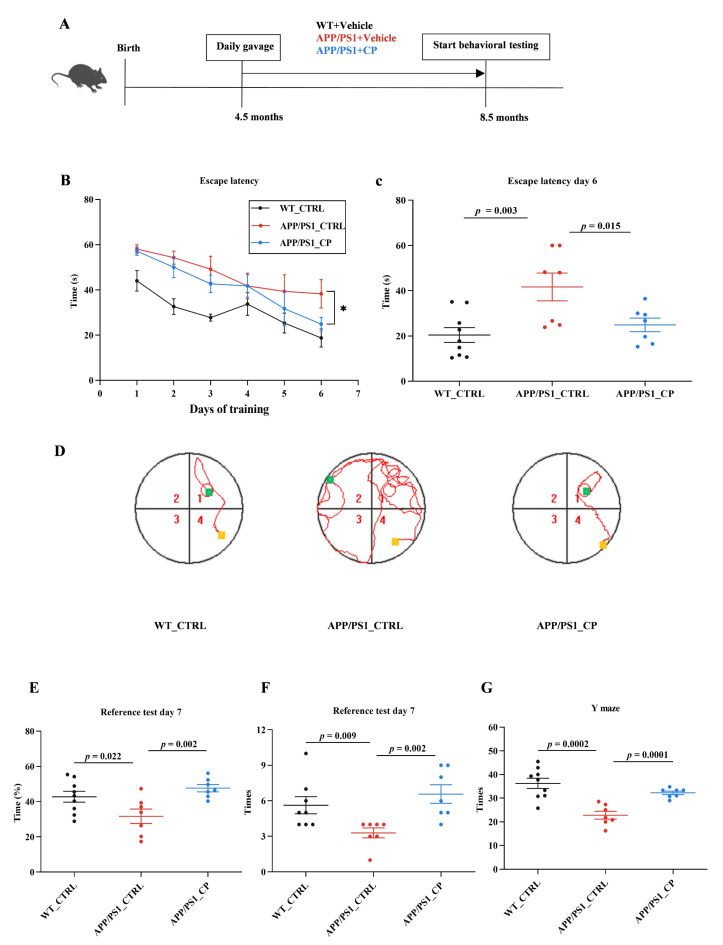
Daily gavage of capsaicin in APP/PS1 mice for 4-month cognitive deficit rescues. (**A**) Dietary capsaicin began at 4.5 month and continued for 4 months (WT_CTRL: *n* = 9; APP/PS1_CTRL: *n* = 7; APP/PS1_CP: *n* = 7). (**B**,**C**) Both WT and APP/PS1 mice showed learning abilities during the training phase. APP/PS1_CTRL mice showed higher escape latencies during acquisition on day 6 than other groups. (**D**) Representative swimming paths on day 6 of training (Yellow and green separately represent the place of origin and terminal; red line represents swimming paths). (**E**) WT_CTRL mice and APP/PS1_CP mice significantly preferred the target quadrant compared with APP/PS1_CTRL mice. (**F**) The number of mice crossing virtual platforms in the MWM test. (**G**) The Y maze test was used to assess spatial working memory in all animals. All data are the mean ± SEM and the minimum significance value was considered as *p* < 0.05. * indicates significant difference.

Following 4 months of either a capsaicin-enriched or vehicle diet (Figure 1A), all mice had their cognitive functions evaluated by using two neurobehavioral tests; the Morris water maze (MWM) test and Y maze test. We assessed the long-term working memory using the MWM test. It was shown that the escape latency of all groups decreased gradually for the duration of the 6-day acquisition, which declined more quickly than vehicle-treated APP/PS1 mice, and was similar to WT mice (Figure 1B). There was a significant effect between capsaicin-treated APP/PS1 mice and vehicle-treated animals on day 6 (*p* < 0.05) (Figure 1C), and capsaicin-treated APP/PS1animals needed a decrease in latency in order to load the platform (Figure 1C,D). On probe trial day 7, capsaicin-treated APP/PS1 mice needed more time to reach the escape platform in the target quadrant during the acquisition period, and they crossed a higher number of platform areas than vehicle-treated animals (*p* < 0.05) (Figure 1E,F). Above all, it can be concluded that capsaicin-treated APP/PS1 mice demonstrated a decline in the escape latency, a significant preference in the target quadrant, and a higher number of platform area crosses compared to vehicle-treated APP/PS1 mice. 

To assess short-term working memory, the Y maze test was performed in all groups. The result revealed that capsaicin-treated APP/PS1 animals had a higher occurrence number of entering the novel arm compared to vehicle-treated animals (*p* < 0.05) (Figure 1G). Capsaicin-treated APP/PS1 mice preferred the novel arm significantly more than vehicle-treated APP/PS1 animals. Overall, these data confirmed that capsaicin-rich diets can rescue impairments and cognitive disorders in APP/PS1 mice.

### 3.2. Capsaicin Reduced the Deposit of Amyloid Plaque in the Hippocampus and Cortex of APP/PS1 Mice

**Figure 2:**
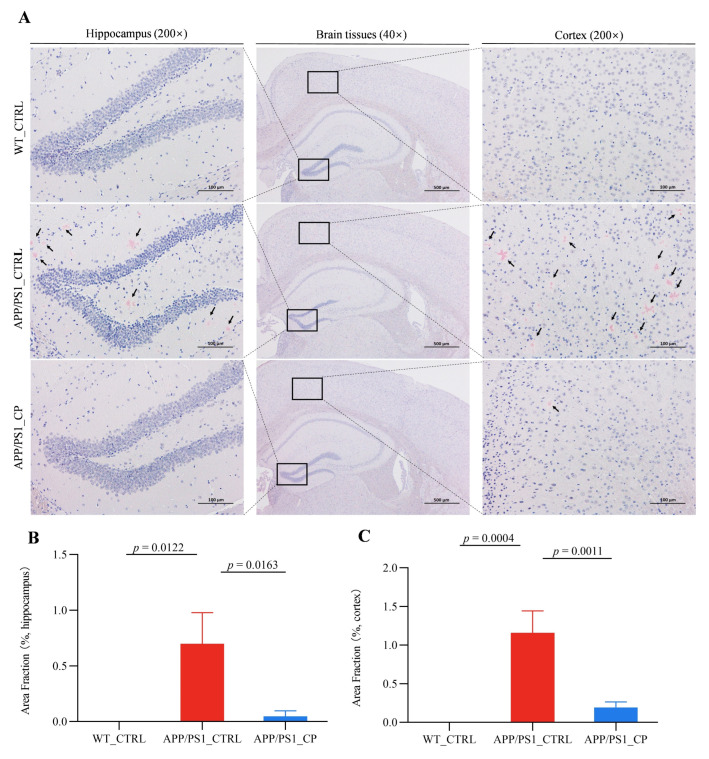
Capsaicin reduces β-amyloid plaques deposits of brain tissues in APP/PS1 mice (all groups: *n* = 4). (**A**) Representative images of brain tissues using Congo red staining in all mice (arrows label Aβ plaques). (**B**,**C**) represent the deposits of β-amyloid plaques in the hippocampus and cortex. Results are mean ± SEM. The minimum significance value was considered as *p* < 0.05.

The aggregation of amyloid plaque in the brain tissues is the pathogenesis of AD [24]. We performed Congo red staining to assess the deposits of amyloid plaque in the hippocampus and cortex of all animals. APP/PS1 mice had a significantly higher amyloid plaque deposits both in the hippocampus and cortex than WT animals (Figure 2A), and the capsaicin diet significantly reduced Aβ aggregations in the hippocampus and cortex. Specifically, capsaicin reduced the total Aβ deposits by 92.85% and 83.62% in the hippocampus and cortex of APP/PS1animals, respectively (Figure 2B,C).

### 3.3. Capsaicin Increased the Relative Abundance of Phylum Verrucomicrobiata, Genus Akkermansia, and Species A. Muciniphila in APP/PS1 Mice

**Figure 3:**
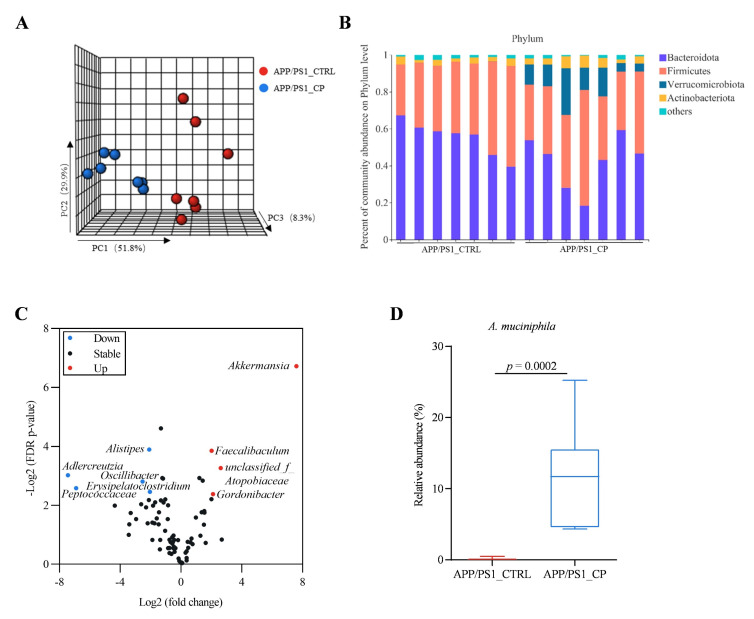
Relative abundance of gut microbiota in mice individuals (all groups: *n* = 7). (**A**) Bray–Curtis-based principal component analysis (PCA) plot of gut microbial communities in APP/PS1 mice. (**B**) Bar plot displaying changes in relative abundances at the phylum level in APP/PS1 mice treated with vehicle or capsaicin. (**C**) Volcano plot displaying differences in gut microbiota between APP/PS1_CTRL and APP/PS1_CP. (**D**) Comparison of the change in *A. muciniphila* between APP/PS1_CTRL and APP/PS1_CP. Results are mean ± SEM. The minimum significance value was considered as *p* < 0.05.

To determine whether capsaicin had an effect on the gut microbiota, the 16S sequencing processes for fecal samples were performed. Principal component analysis (PCA) showed a clear difference between the microbial communities of capsaicin-treated and vehicle-treated APP/PS1 mice (Figure 3A). Different alpha-diversity indices, including Shannon (*p* = 0.0413), Simpson (*p* = 0.0295), Chao (*p* = 0.4616) and Ace (*p* = 0.3999), indicated that capsaicin significantly decreased gut bacterial alpha diversity, which was related with the antibacterial activity of capsaicin (Figure S1A). Comparing the relative abundances of the bacterial communities between capsaicin-treated and vehicle-treated APP/PS1 mice, we found changes in the bacterial phylum levels (Figure 3B). The capsaicin-treated APP/PS1 mice harbored a higher relative abundance of Verrucomicrobiota than vehicle-treated animals (*p* < 0.0001). Moreover, we also observed that capsaicin diet increased the relative abundance of bacterial genera, including *Akkermansia*, *Faecalibaculum*, *Unclassified_f_Atopobiaceae*, *Gordonibacter* and decreased the relative abundance of bacterial genera, including *Adlercreutzia*, *Peptococcaceae*, *Alistipes*, *Oscillibacter*, *Erysipelatoclostridium* (Figure 3C). The abundance of phylum Verrucomicrobiota, genus *Akkermansia,* and species *A. muciniphila* (all of which comprise Gram-negative bacterial groups) in the APP/PS1_CP group significantly increased compared to the APP/PS1_CTRL group (*p* = 0.0002) (Figure 3D). The results implied that capsaicin has a profound effect on the composition of microbiota.

Studies have found that the relative abundance of *A. muciniphila* in APP/PS1 mice decreased with age [25]. Moreover, recent evidence suggests that supplementation with *A. muciniphila* obviously reduced amyloid aggregation in the cerebral cortex and improved cognitive deficits and in AD mice [26]. In the present study, the higher abundance of species *A. muciniphila* in APP/PS1_CP versus APP/PS1_CTRL animals is significant (Figure 3D), which is in agreement with previous data.

### 3.4. Capsaicin Upregulated Tryptophan Metabolism and Downregulated Lipid Metabolism in APP/PS1 Mice

**Figure 4:**
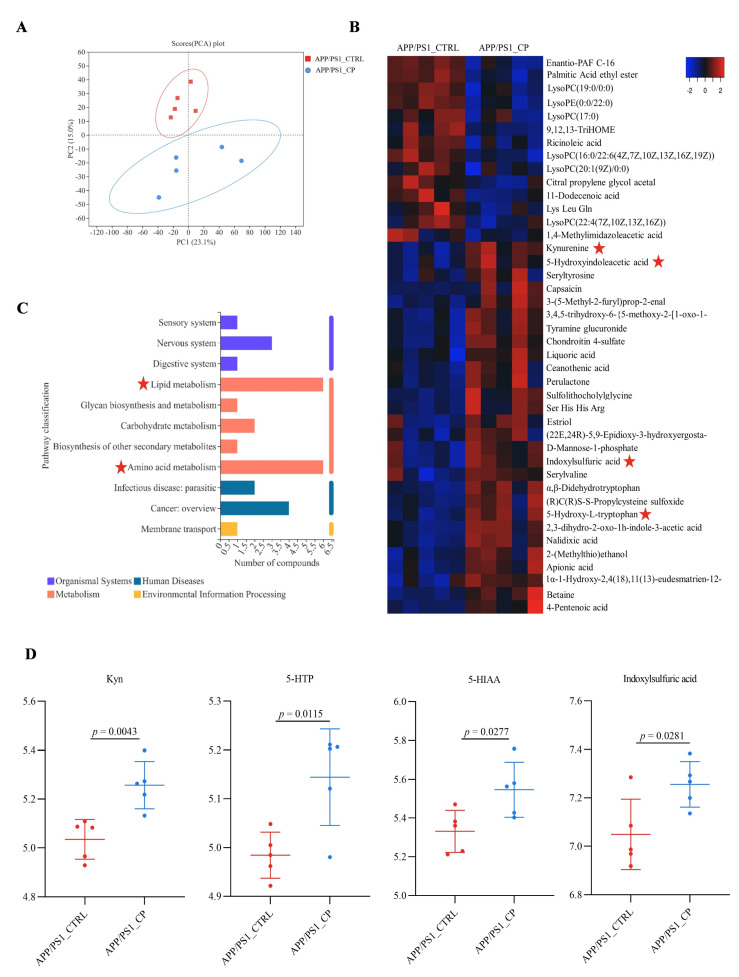
APP/PS1 mice show significant effects in serum metabolites that are normalized by capsaicin (all groups: *n* = 5). (**A**) Principal component analysis (PCA) was used to measure the metabolite profiles in serum samples. (**B**) Hierarchical clustering analysis of the top 50 significant metabolites by ANOVA showed a significant difference of APP/PS1_CP on serum metabolism compared to APP/PS1_CTRL. (**C**) Comparison of the change in serum metabolic pathways between APP/PS1_CTRL and APP/PS1_CP. (**D**) Significant effects of capsaicin treatment were related to Trp metabolism in APP/PS1 mice. Results are mean ± SEM. The minimum significance value was considered as *p* < 0.05.

Serum metabolites link the microbiome to its host by regulating metabolism [27]. Therefore, serum metabolome processes were performed using LC/MS-MS. The PCA analysis showed that serum metabolic profiles in capsaicin-treated APP/PS1 mice were significantly different from vehicle-treated APP/PS1 mice (Figure 4A). Forty-two metabolites were specifically altered in the serum of the APP/PS1_CP group compared with the APP/PS1_CTRL group (Figure 4B).

The altered metabolites were involved in various metabolic pathways. Multiple previous studies showed that short-chain fatty acids, bile acids, and tryptophan (Trp) metabolites are the three main metabolic pathways related to host–microbiota interactions [28]. Amino acid metabolism and lipid metabolism were the main pathways between APP/PS1_CP mice and APP/PS1_CTRL mice in this study (Figure 4C). Specifically, capsaicin upregulated amino acid metabolism and downregulated lipid metabolism in APP/PS1 mice.

Focusing on overlapping metabolites, we found four metabolites linked with tryptophan metabolism, which is the essential aromatic amino acid. The relative abundances of four metabolites Kyn (*p* = 0.0043), 5-HIT (*p* = 0.0115), 5-HIAA (*p* = 0.0277), and indoxylsulfuric acid (*p* = 0.0281) increased significantly in APP/PS1_CP mice compared with APP/PS1_CTRL mice (Figure 4D). Lysophosphatidyl cholines (LysoPCs) and lysophosphatidyl ethanolamine (LysoPE) are linked with lipids metabolism. Specifically, LysoPC (17:0) (*p* = 0.0289), LysoPC (22:4(7Z, 10Z, 13Z, 16Z)) (*p* = 0.0188), LysoPC (16:0/22:6(4Z, 7Z, 10Z, 13Z, 16Z, 19Z)) (*p* = 0.0008), LysoPC (19:0/0:0) (*p* = 0.0036), LysoPC (20:1(9Z)/0:0) (*p* = 0.0331), and LysoPE (0:0/22:0) (*p* = 0.001) decreased significantly in capsaicin-treated APP/PS1 mice (Figure S2).

### 3.5. The Correlation between Gut Microbiota and Serum Metabolites

**Figure 5:**
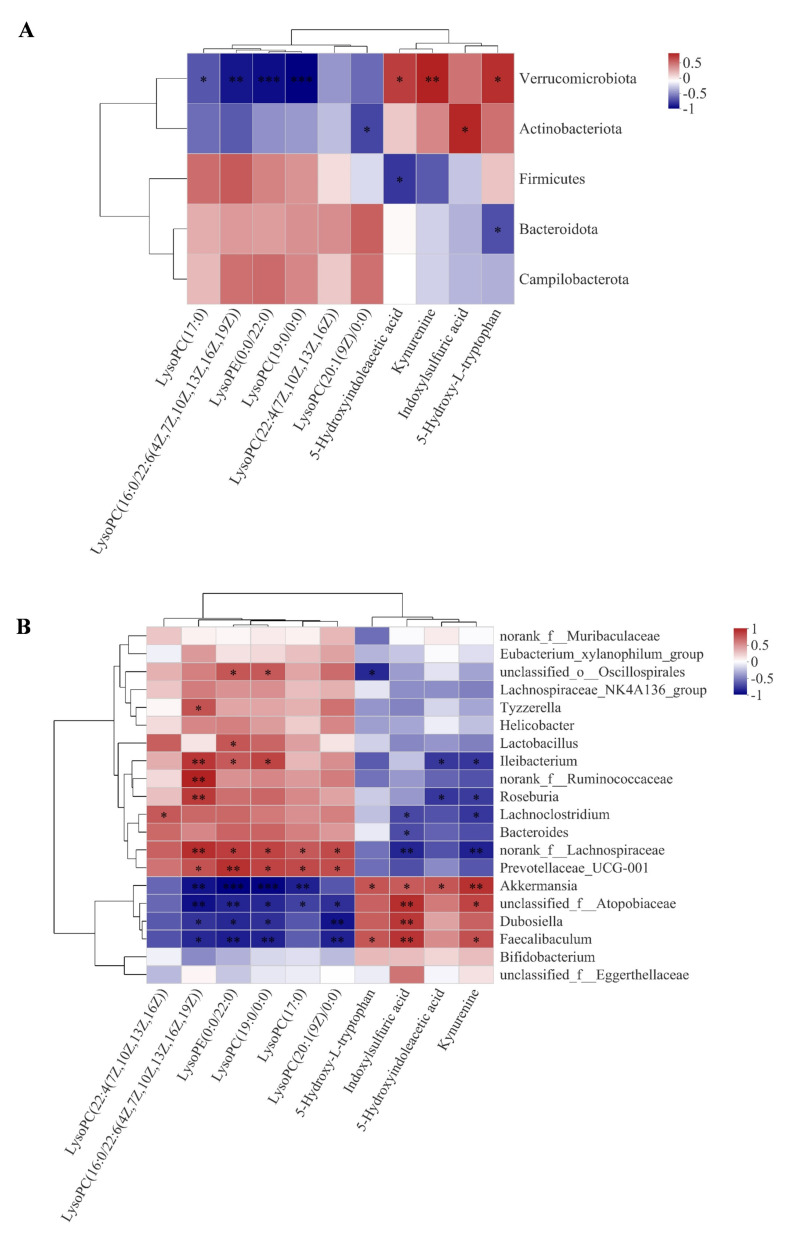
Spearman’s correlation analysis of gut microbiota and serum metabolites. Correlation between the gut microbiome and serum metabolites at (**A**) the phylum level and (**B**) the genus level between APP/PS1_CTRL mice and APP/PS1_CP mice (* means *p* < 0.05; ** *p* < 0.01 and *** *p* < 0.001).

Spearman’s correlation analyses between gut bacteria and serum metabolites were performed to investigate the links between both sides. At the phylum level, we observed that Verrucomicrobiota significantly changed microbiota in capsaicin-treated APP/PS1 mice (*p* = 0.0002) (Figure 5A). Verrucomicrobiota had positive correlations with Kyn, 5-HIAA, and 5-HIT. Kyn includes the three metabolites of tryptophan metabolisms. Moreover, Verrucomicrobiota was negatively correlated with lysophosphatidyl cholines (LysoPCs), such as LysoPC (17:0), LysoPC (16:0/22:6(4Z, 7Z, 10Z, 13Z, 16Z, 19Z)), LysoPC (19:0/0:0), and lysophosphatidyl ethanolamine (LysoPE) (0:0/22:0), which are the metabolites of lipid metabolisms. The intake of capsaicin diets significantly increased the relative abundance of Verrucomicrobiota, which may upregulate Trp metabolisms and downregulate lipid metabolisms in APP/PS1 mice.

At the genus level, metabolic association heatmap analyses revealed correlations between the levels of gut microbiota and serum metabolites (Figure 5B). *Akkermansia*, *Unclassified_f_Atopobiaceae*, and *Faecalibaculum*, which were the significantly increased microbiota in capsaicin-treated APP/PS1 mice compared to vehicle-treated APP/PS1 mice (Figure 2C), displayed positive correlations with Trp metabolites and negative correlations with lipid metabolites.

*Akkermansia* had a positive correlation with metabolites such as 5-HIAA, Kyn, 5-HIT and indoxylsulfuric acid, but it had a negative correlation with metabolites such as LysoPC (17:0), LysoPC (16:0/22:6(4Z, 7Z, 10Z, 13Z, 16Z, 19Z)), LysoPC (19:0/0:0) and LysoPE (0:0/22:0). *Unclassified_f_Atopobiaceae* had a positive correlation with metabolites such as Kyn and indoxylsulfuric acid but a negative correlation with metabolites such as LysoPC (17:0), LysoPC (20:1(19Z)/0:0), LysoPC (19:0/0:0), LysoPC (16:0/22:6(4Z, 7Z, 10Z, 13Z, 16Z, 19Z)), and LysoPE (0:0/22:0). *Faecalibaculum* had a positive correlation with metabolites such as Kyn, 5-HIT, and indoxylsulfuric acid, but it had a negative correlation with metabolites such as LysoPC (17:0), LysoPC (20:1(19Z)/0:0), LysoPC (19:0/0:0), LysoPC (16:0/22:6(4Z, 7Z, 10Z, 13Z, 16Z, 19Z)), and LysoPE (0:0/22:0).

## 4. Discussion

Numerous studies have discovered that interactions between the microbiome’s metabolites and hosts influenced how the gut microbiome and microbial metabolites regulated brain function and behavior, which may have an impact on the development of AD [29,30,31]. For example, GF mice displayed the impairment of immune responses and the pathogenesis of AD by modulating the number of cells and the maturity of microglia, which resulted in impaired spatial and working memory [32,33]. Antibiotics, such as streptozotocin and ampicillin, can damage the balance of gut bacteria and worsen the development of AD [34]. The administration of ampicillin in rats disrupted the gut microbiota, elevated serum corticosterone, and, thus, impaired spatial memory [33]. APP/PS1 transgenic mice treated with a cocktail of antibiotics (ABX) had increased levels of neuroinflammatory and cytokine-related genes [35]. Therefore, the adjustment of gut microbiota composition maybe a considerable treatment for AD by supplying with probiotics or designing individual diets.

The dietary interventions have a close association with the occurrence of AD by regulating gut microbiota [35]. For instance, dietary ω-3 PUFAs significantly balanced gut microbiota compositions in mice and reduced the risk of AD [36,37]. Coffee drinkers had a lower occurrence of AD compared to individuals with persons drinking no or little coffee, and this is because the fiber and polyphenols in coffee beans regulated the number and compositions of gut microbiota [38,39]. The daily intake of dietary fruits and vegetables balances the gut microbiota, and this may play important roles in AD control [40,41]. The pungent capsaicin molecule is one of the bioactive products in chili pepper, and it is consumed when ingesting vegetables and spices. In this study, the dietary consumption of capsaicin significantly increased the relative abundance of phylum Verrucomicrobiota, genus *Akkermansia*, and species *A. muciniphila* in APP/PS1 mice and ameliorated cognitive deficits (Figure 1 and Figure 3).

*muciniphila* was isolated from the human intestine and characterized before two decades. Multiple studies linked numerous diseases with either a lack or decreased abundance of this bacterium [42,43]. Although some studies have observed that AD patients had a higher percentage of *A. muciniphila* [44,45], intervention experiments clearly reported beneficial effects of this bacterium in the pathology of AD [26]. Alzheimer’s disease is a disorder and Aβ plaques are pathological hallmarks. By contrast, clinical studies report the protective effects of *A. muciniphila*. Moreover, capsaicin-enriched diets significantly increased the relative abundance of *A. muciniphila* in APP/PS1 mice (Figure 3D). On the basis of promising initial studies, *A. muciniphila* contributed to tryptophan secretion, and increased *A. muciniphila* abundance was correlated with an improved metabolic profile [46]. The upregulated metabolic factors of Trp crossed the blood–brain barrier (BBB) and provided neuronal protection, such as plaques clearness and cognitive benefits [47].

**Figure 6:**
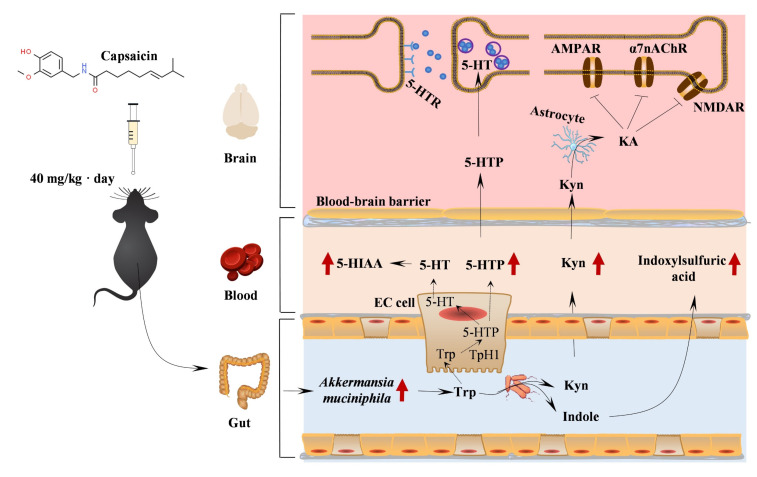
The interaction between the microbiota and the microbiota–gut–brain axis in APP/PS1 mice and capsaicin intervention. Dietary capsaicin can directly change gut microbiota compositions and significantly enhance the relative abundance of *A. muciniphila*, which contributed to Trp secretion. Almost all free Trps are involved in the Kyn pathway (KP) related to Trp degradation and the gut microbiota influence the kynurenine-producing indoleamine-2,3-dioxygenase (IDO) pathway. Kynurenine amino transferase in astrocytic cells can transfer from Kyn to kynurenic acid (KA), which regulates cognition and behavior using three receptors, including the α7-nicotinic receptor (α7nAChR), N-methyl-d-aspartate receptors (NMDARs), and α-amino-3-hydroxy-5-methyl-4-isoxazolepropionic acid receptors (AMPARs) [28]. The peripheral production of 5-HT and 5-HTP by enterochromaffin (EC) cells is also affected by the gut microbiota. 5-HTP, transported across the blood–brain barrier, is further metabolized into 5-HT, which exhibits neurobiological functions using 5-HT receptors in AD [48]. Gut microbial tryptophanase converts Trp into indole, and then it enters the host portal circulation and is transferred into indoxylsulfuric acid in the liver [49]. The red arrows represent the higher abundance of gut microbiota and increased serum metabolites in capsaicin-treated APP/PS1 mice.

The metabolism of Trp is one of three currently most studied categories of metabolites in the interconnection between the host and its microbiome [28]. Amino acid metabolism and lipid metabolism were two main metabolism pathways in the serum metabolic profile (Figure 4C). Moreover, *Peptococcaceae* and *Alistipes* had decreased relative abundances in capsaicin-treated mice. Thus, amino acids were the main factors relative to metabolism. As shown in Figure 6, Kyn, 5-HTA, 5-HIAA and indoxylsulfuric acid, which are generated from the three pathways of Trp metabolism in the gastrointestinal tract, had significantly higher level than capsaicin-free control mice. This result suggests that the capsaicin-enriched diet could improve Trp metabolism in APP/PS1 mice. Multiple previous studies indicated that almost all free Trps were involved in the Kyn pathway (KP) of Trp metabolism [47]. Moreover, nearly 60% of Kyns, transported across the blood–brain barrier, are distributed in the central nervous system (CNS). According to Kyns pathways in the CNS, astrocytes are equipped to secrete kynurenic acid (Kyna), which provides neuronal protection. On the basis of behavioral tests and the β-amyloid clearance of brain tissues (Figure 1 and Figure 2), the neuroprotective Kyna was the main metabolic factor of Kyn, which was utilized by astrocytes in the brain tissues.

Long-chain fatty acid is inversely associated with the pathogenesis of obesity [50]. Obesity, strongly related to AD in aging, is the most common metabolic disorder. Metabolic disorders caused by brain-tissue atrophy, reduced gray and white matter volume, alteration in the electrical characteristics of nerve tissue, a reduction in hippocampal neurogenesis and impairment in the proliferation and differentiation of neuroprogenitor cells (NPCs) into neurons, neuronal death, decreased synaptic plasticity, and impairment of BBB integrity induce cognitive impairment and AD [51]. Dietary capsaicin activated its receptor, transient receptor potential vanilloid 1 (TRPV1), and converted adipose tissues from white to brown, which contributed to reducing obesity [52]. Capsaicin has been reported its anti-obesity effect on high fat diet-fed mice by altering gut microbiota compositions, increasing the relevant abundance of genus *Akkermansia* [53]. Furthermore, numerous studies identified a close association between *A. muciniphila* and obesity. The daily intervention of pasteurized *A. muciniphila* reduced food energy efficiency and mitigated diet-induced obesity in diet-induced obese mice [54]. *A. muciniphila* regulated L-aspartate metabolism and improved fatty liver associated with metabolic dysfunction [55]. *A. muciniphila* mitigated metabolism-induced inflammation and prevented obesity-related atherosclerosis by in Apoe^-/-^ mice [56]. In this research study, we observed that capsaicin significantly increased the relative abundance of *A. muciniphila* and decreased the level of long-chain fatty acids, such as LysoPC (17:0) (*p* = 0.0289), LysoPC (22:4(7Z, 10Z, 13Z, 16Z)) (*p* = 0.0188), LysoPC (16:0/22:6(4Z, 7Z, 10Z, 13Z, 16Z, 19Z)) (*p* = 0.0008), LysoPC (19:0/0:0) (*p* = 0.0036), LysoPC (20:1(9Z)/0:0) (*p* = 0.0331), and LysoPE (0:0/22:0) (*p* = 0.001) in APP/PS1 mice (Figure S2). Our results are consistent with those of previous studies.

## 5. Conclusions

In this study, we observed that capsaicin rescued the behavioral cognitive deficits of APP/PS1 mice. Congo red staining showed that capsaicin decreased β-amyloid deposits of the brain tissues in APP/PS1 mice. Gut microbiota profiling indicated that the capsaicin-treated mice harbored higher relative abundances of *Akkermansia*, *Faecalibaculum*, *Unclassified_f_Atopobiaceae*, and *Gordonibacter*, while the vehicle-treated APP/PS1 mice harbored higher relative abundances of *Adlercreutzia*, *Peptococcaceae*, *Alistipes*, *Oscillibacter*, and *Erysipelatoclostridium*. Notably, *A. muciniphila* was observed to be significantly increased in the capsaicin-treated mice. The metabolome profiling of serum samples showed that capsaicin-treated mice had a higher level of Trp metabolism and a lower level of lipid metabolism compared to vehicle-treated APP/PS1 mice. The evidence of *A. muciniphila* and Trp metabolites highlighted that capsaicin-enriched diets may mitigate the incidence and development of AD by changing gut microbiome and serum metabolome, and *A. muciniphila* and Trp metabolism may be possible approaches for ameliorating AD.

## Data Availability

Sequencing reads and the genome assembly are available in NCBI under bioproject PRJNA895588. The fecal metabolite data are available in MetaboLights under MTBLS6342.

## Supplementary Materials

The following supporting information can be downloaded at: www.mdpi.com/article/10.3390/nu15010118/s1, Figure S1: Relative abundance of gut microbiota in APP/PS1 mice (APP/PS1_CTRL: *n* = 7; APP/PS1_CP: *n* = 7). (A) Alpha diversity analysis of gut bacterial diversity and richness from different mouse groups. (B) Bar plot showing changes in the relative abundance of different bacterial classes at the genus level in APP/PS1 mice treated with vehicle or capsaicin; Figure S2: Significant effects of capsaicin treatment were noted with respect to lipid metabolism in APP/PS1 mice (all groups: *n* = 5).
