# Supplementary material for: Gut Microbiome and Serum Metabolome Profiles of Capsaicin with Cognitive Benefits in APP/PS1 Mice
Source: Nutrients. 2022 Dec 27;15(1):118. doi: 10.3390/nu15010118 (PMC9823564; doi:10.3390/nu15010118)
Supplement: Supplementary file 1 [file nutrients-15-00118-s001.zip › nutrients-2078642-supplementary.pdf]

Supplementary materials

Gut microbiome and serum metabolome profiles of capsaicin with cognitive benefit in APP/PS1 mice

Jun Li[1], Xiaojun Liao[1], Xuedong Yin[2], Zimeng Deng[1], Guangfen Hu[2], Weiwei Zhang[1], Feng Jiang[2], Liang Zhao[1,*]

[1] National Engineering Research Center for Fruit & Vegetable Processing, Key Laboratory of Fruit & Vegetable Processing, Ministry of Agriculture and Rural Affairs, Engineering Research Centre for Fruits and Vegetables Processing, Ministry of Education, Beijing Key Laboratory for Food Non-thermal Processing, College of Food Science and Nutritional Engineering, China Agricultural University, Beijing 100083, China

[2] Guizhou Guisanhong Food Company Limited, Zunyi 563000, China

*Corresponding author:

Mailing address: College of Food Science & Nutritional Engineering, China Agricultural University, No. 17 Qinghua East Road, Haidian District, Beijing 100083, China (Liang Zhao)

E-mail addresses: zhaoliang1987@cau.edu.cn (Liang Zhao),

A

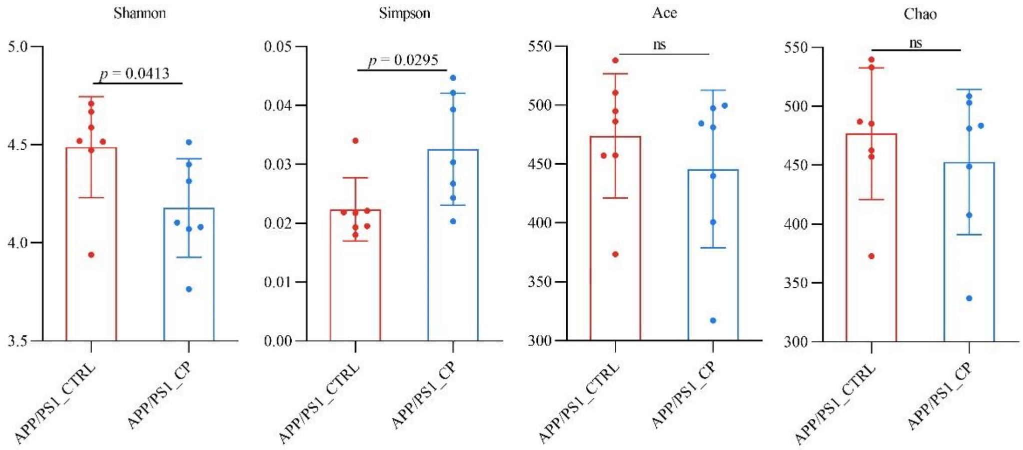

B

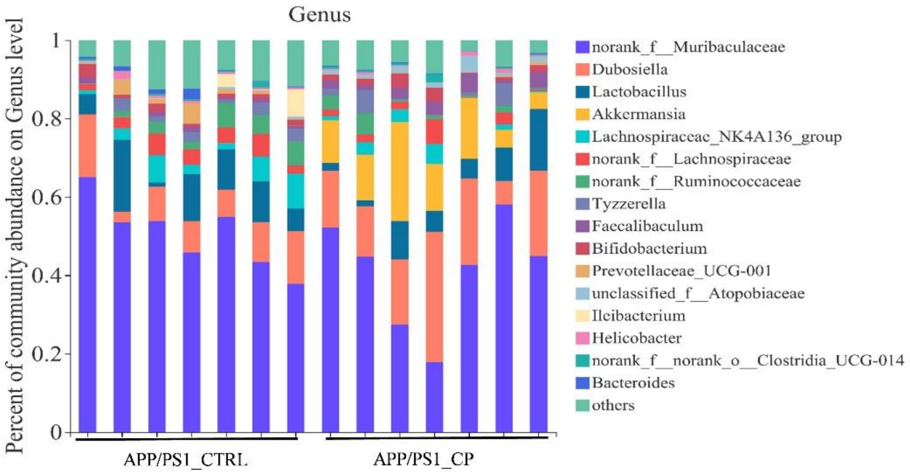

Figure S1. Relative abundance of gut microbiota in APP/PS1 mice (APP/PS1_CTRL: $n = 7$; APP/PS1_CP: $n = 7$). (A) Alpha diversity analysis of gut bacterial diversity and richness from different mouse groups. (B) Bar plot showing changes in relative abundance of different bacterial classes at the genus level in APP/PS1 mice treated with vehicle or capsaicin. All data are the mean ± SEM and the minimum significance value was considered as $p < 0.05$.

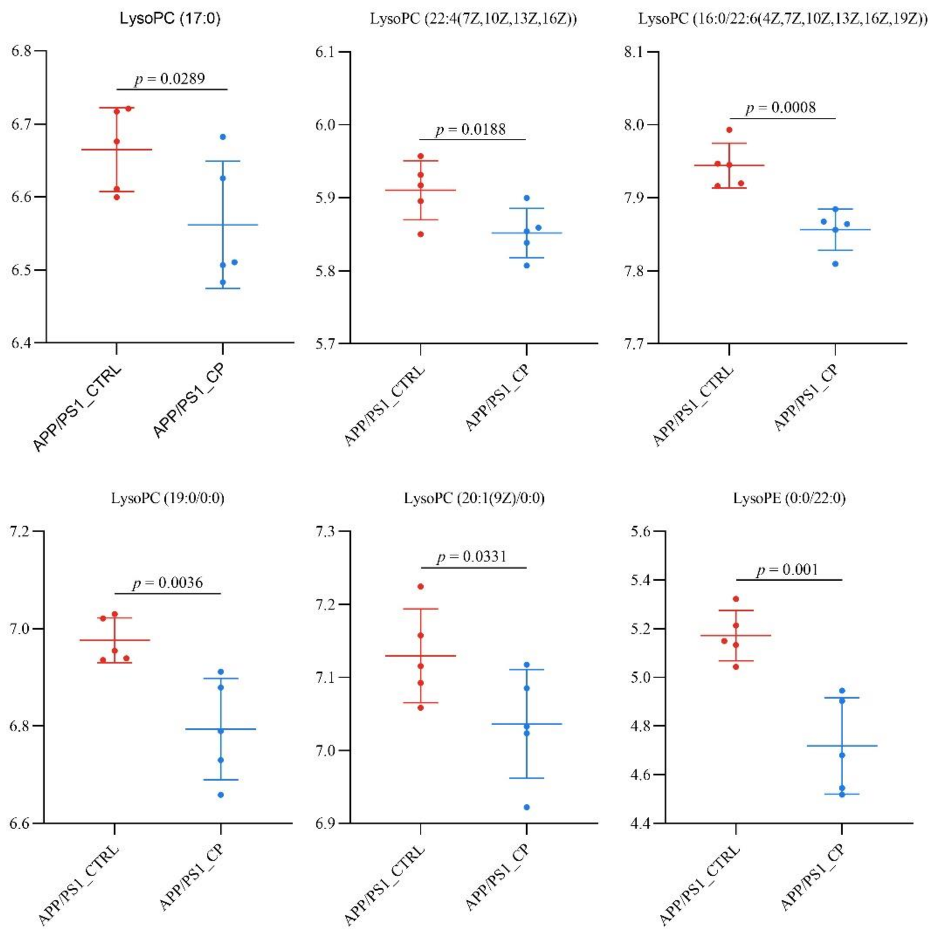

Figure S2. Significant effects of capsaicin treatment were noted with respect to lipid metabolism in APP/PS1 mice (all groups: $n = 5$). All data are the mean ± SEM and the minimum significance value was considered as $p < 0.05$.
